# Injury mortality report for São Paulo State, 2003

**DOI:** 10.1590/S1516-31802007000300003

**Published:** 2007-05-03

**Keywords:** Mortality rate, Violence, Accident prevention, Homicide, Traffic accidents, Coeficiente de mortalidade, Violência, Prevenção de acidentes, Homicídio, Acidentes de trânsito

## Abstract

**CONTEXT AND OBJECTIVE:**

Injuries are an important public health issue in the State of São Paulo. Each year around 32,000 people are killed and 180,000 are hospitalized due to injury. The aim of this work was to analyze fatal injury data to provide an overview on mortality due to injuries in the State of São Paulo in 2003, the most recent year for which data are available.

**DESIGN AND SETTING:**

Population-based descriptive study carried out in the State Health Department of São Paulo.

**METHODS:**

Data from 31,032 deaths due to injury were analyzed. The dataset was from the Mortality Information System (SIM/DATASUS). The data were stratified by sex, age, intent and mechanism of injury. Unadjusted and age and sex-specific rates were calculated per 100,000 population.

**RESULTS:**

The unadjusted injury mortality rate was 80.2/100,000 (140.2/100,000 for males and 22.4/100,000 for females). The highest rates were found for males and among young and old people. A greater proportion of intentional injuries than of unintentional deaths resulted in death (49.73% and 39.7%, respectively). Homicides were the leading cause, 44.6% overall (35.8/100,000), followed by motor vehicle traffic, 22.3% overall (17.9/100,000). Firearms played an important role among homicide deaths. Intention and mechanism of injuries varied according to sex and age group.

**CONCLUSION:**

These data indicate a need to develop injury prevention strategies, considering the magnitude of the problem and the groups at high risk. Homicides among young people have to be addressed. Motor vehicle traffic injuries play an important role for all ages.

## RESUMO

**CONTEXTO E OBJETIVO:**

No estado de São Paulo as causas externas de morte são um importante problema de saúde pública. A cada ano, cerca de 32.000 pessoas morrem e outras 180.000 são hospitalizadas devido a essas causas. O objetivo deste trabalho foi analisar dados de mortes por causas externas para o estado de São Paulo, 2003.

**TIPO DE ESTUDO E LOCAL:**

Estudo descritivo com base populacional. Realizado no Estado de São Paulo, 2003.

**MÉTODO:**

O universo de 31.032 mortes por causas externas foi analisado. O banco de dados utilizado foi o Sistema de Informações de Mortalidade (SIM/DATASUS). Os dados foram estratificados por sexo, idade, intencionalidade e mecanismo da causa externa. As taxas brutas foram calculadas por 100.000 habitantes.

**RESULTADOS:**

A taxa de mortalidade encontrada foi 80,2/100.000 (140,2/100.000 para os homens e 22,4/100.000 para as mulheres). Taxas mais altas foram encontradas entre os homens, os jovens e os idosos. As mortes classificadas como intencionais foram 49,7%, enquanto que as não intencionais foram 39,7%. Homicídios foram a principal causa, 44,6% do total (35,8/100.000), seguidas pelos acidentes de transporte terrestre, 22,3% do total (17,9/100.000). Armas de fogo tiveram um importante papel nas mortes por homicídios. A intencionalidade e o mecanismo da causa externa variaram de acordo com a idade da vítima.

**CONCLUSÕES:**

Os resultados indicam a necessidade de desenvolver estratégias para a prevenção do problema, que devem levar em conta as diferenças existentes entre as faixas etárias no que diz respeito à intencionalidade e mecanismo da lesão. Especial atenção deve ser dada aos homicídios e aos acidentes de transporte.

## INTRODUCTION

Injuries have been shown to cause a significant health burden on many countries around the world,^1^ including Brazil. Compared with other countries, Brazilian injury mortality rates are very high.^2,3^ This problem reflects many characteristics of the Brazilian population, including personal and social factors. According to the Brazilian Institute for Geography and Statistics (IBGE), life expectancy is increasing in Brazil (71.3 years for 2003) but it has been held back by two or three years because of premature mortality due to injuries.^4^

São Paulo is the most populous state in Brazil (population for 2004: approximately 39 million) and studies have shown that injuries are a huge problem, with an impact on health and economics.^5,6^ Each year around 32,000 people are killed and 180,000 are hospitalized because of an injury. In 2003, injuries were the third biggest cause of death and were the leading cause for people aged one to 44 years.

Although the term injury is widely used in the international literature, in Brazil it is more common to use *external cause* or *accidents and violence* to express the notion of an injury. An *injury* is defined by the World Health Organization as caused by acute exposure to physical agents such as mechanical energy, heat, electricity, chemicals and ionizing radiation, interacting with the body in amounts or at rates that exceed the threshold of human tolerance. In some cases (for example, drowning and frostbite), injuries results from the sudden lack of essential agents such as oxygen or heat.^7^ Among injuries, some causes are classified as unintentional (transport accidents, falls, drowning etc.), other causes are intentional (suicide, homicide or legal intervention) and there are also undetermined events (in which it was not possible to determine whether the event was intentional or unintentional).

## OBJECTIVE

This report was developed to raise awareness of the impact of injuries on health and to call on health professionals to play a wider role in preventing this problem. This was a based-population descriptive study, in which the goal was to provide an overview of fatal injury data for the State of São Paulo in 2003, the most recent year for which data are available.

## METHODS

The data in this report are based on death certificates. The data source was the Mortality Information System (SIM/DATASUS) operated by Brazil's Ministry of Health. The case definition adopted for mortality was death certificates on which the underlying cause of death was classified in the International Classification of Diseases, Tenth Revision (ICD-10), chapter XX.^8^ This report presents data on external causes of death due to injury death by using the ICD-10 mortality matrix.^9,10^ This matrix was developed by the Injury Control and Emergency Health Services (ICEHS) section of the American Public Health Association and the International Collaborative Effort (ICE) on Injury Statistics, with the aim of enabling international comparisons. The present report is the first injury mortality report in Brazil to use this matrix.

The causes of death were classified according to intent and mechanism. The injuries causing mortality were classified as intentional (suicide, homicide or legal intervention), unintentional (motor-vehicle accidents, falls, drowning or other accidental causes) and undetermined. The data were stratified by sex, age and type of injury. Unadjusted and age and sex-specific rates were calculated per 100,000 of population. Most of the analyses were performed according to age groups in order to highlight the differences in injury mortality patterns between these age groups. The population data for the State of São Paulo were obtained from the Brazilian Institute for Geography and Statistics (Instituto Brasileiro de Geografia e Estatística, IBGE).

## RESULTS

In 2003, a total of 31,032 persons died from an injury in the State of São Paulo: 26,591 were males (86.0%) and 4,432 were females (14.0%). Adolescents and young adults aged 15 to 29 years accounted for 42.3% of the injury victims. Intentional deaths were predominant, accounting for 49.7% overall, while unintentional deaths accounted for 37.7% and undetermined causes for 3.6% of deaths. The unadjusted injury rate for the whole population was 80.2/100,000 and the male rate (140.2/100,000) was 6.3 times the female rate (22.4/100,000). The death rate due to injury ranged from a minimum of 13.1/100,000 among children and pre-adolescents aged 0 to 14 years to a maximum of 120.8/100,000 among adolescents and young adults aged 15 to 29 years.

### Age, intent and type of injury

The data were analyzed in five different age-group patterns (Tables 1 and 2). The first group was the population from 0 to 14 years of age, i.e. children and younger adolescents. This age group showed the lowest injury rates of all the age groups. The unintentional component predominated: 73.2% overall. The unadjusted mortality rate was 13.5/100,000 for all injuries and the leading cause was motor vehicle traffic accidents (MVT) (4.1/100,000). Among these traffic deaths, 42.4% were as pedestrians. Suffocation was in second position (2.1/100,000), followed by drowning (1.8/100,000). Among the intentional injuries, homicides were the leading cause, mostly by firearms.

**Table 1.** Injury mortality by age group and intent and selected injury mechanisms (number and rate/100,000), São Paulo state, 2003

	Age group (years)												
Intent and mechanism	0 to 14		15 to 29		30 to 44		45 to 59		60 and over		Unknown	Total	
	n	Rate	n	Rate	n	Rate	n	Rate	v	Rate	n	n	Rate
All unintentional	1,030	10.1	3,492	32.2	2,883	32.5	2,101	39.5	2,057	59.6	140	11,703	30.2
Motor vehicle traffic	417	4.1	2,411	22.2	1,871	21,1	1,251	23.5	894	25.9	101	6,945	17.9
Pedestrian	194	1.9	438	4.0	565	6.4	434	8.2	465	13.5	78	2,174	5.6
Falls	60	0.6	96	0.9	275	3.1	304	5.7	704	20.4	3	1,442	3.7
Drowning	180	1.8	469	4.3	228	2.6	137	2.6	49	1.4	36	1,099	2.8
Suffocation	215	2.1	22	0.2	54	0.6	42	0.8	87	2.5	-	420	1.1
All intentional	189	1.9	8,756	80.6	4,308	48.5	1,475	27.8	461	13.4	263	15,452	39.9
Homicides	178	1.7	8,234	75.8	3,827	43.1	1,112	20.9	290	8.4	258	13,899	35.8
Firearms	112	1.1	6,132	56.5	2,477	27.9	613	11.5	112	3.2	124	9.570	24.7
Suicides	11	0.1	522	4.8	481	5.4	362	6.8	171	5.0	5	1,552	4.0
Poisoning	-	-	54	0.5	63	0.7	55	1.0	12	0.3	-	184	0.5
Suffocation	7	0.1	236	2.2	236	2.7	187	3.5	86	2.5	2	754	1.9
Undetermined	157	1.5	747	6.9	919	10.3	635	12.0	1,093	31.7	124	3,675	9.5
All other	-	-	120	1.1	39	0.4	14	0.3	21	0.6	8	202	0.5
**Total**	**1,376**	**13.5**	**13,115**	**120.8**	**8.149**	**91.7**	**4,225**	**79.5**	**3,632**	**105.2**	**535**	**31,032**	**80.2**

*Source: death data are from the Mortality Information System and population data are from the Brazilian Institute for Geography and Statistics (IBGE).*

*Note: Rates for the groups of victims of unknown age were not calculated because the denominator was not available.*

**Table 2.** Injury mortality rates (per 100,000) by intent, State of São Paulo (2003) and selected states in the United States (2001)

	State				
Intent and mechanism	São Paulo	California	District of Columbia	New Mexico	New York
All unintentional	30.2	23.5	38.9	55.7	48.3
Motor vehicle traffic	17.9	11.0	9.4	22.6	8.5
Falls	3.7	3.4	7.5	9.0	5.0
All intentional	39.9	14.6	41.3	26.9	21.4
Homicides	35.8	6.4	34.3	7.1	14.8
Firearms	24.7	4.6	24.6	3.7	3.1
Suicides	4.0	8.2	7.0	19.8	6.6
Poisoning	0.5	0.8	*	4.6	1.7
Suffocation	1.9	2.2	*	4.6	1.7
Undetermined	9.5	0.3	*	0.9	*
**Total**	**80.2**	**38.6**	**82.4**	**83.6**	**48.3**

* *Does not meet standard of reliability or precision.^10^*

The second age group consisted of persons aged 15 to 29 years, i.e. older adolescents and young adults. They showed the highest injury rates in relation to all other age groups: 120.8/100,000. Intentional deaths predominated, representing 66.7% overall, while 5.7% of the deaths in this age group were classified as having undetermined intent. Among the injury mechanisms, homicides were the cause of death of 66.7% of all victims in this age group and the homicide mortality rate reached 75.8/100,000. Firearms played an important role among these deaths, accounting for 74.5% of the homicides. MVT was in second position (22.2/100,000) and the proportion of these MVT events that were as pedestrians (15.5%) was about half of what it was for all other age groups. For this age group, the risk of being a fatal victim of homicide was 3.4 times higher than the risk of being a fatal MVT victim. This age group showed the highest mortality rate due to drowning in relation to all the other age groups.

The analysis of the third age group, persons aged 30 to 44 years, showed a pattern that was similar to the one for people aged 15 to 29 years, but the mortality rate due to firearms was lower. The overall injury mortality rate was 91.7/100,000. Intentional deaths predominated, but the proportion (52.9%) was lower than in the age group from 15 to 29 years; unintentional deaths accounted for 35.4%. The leading cause of these deaths was homicides (43.1/100,000) and 64.7% of these deaths were due to firearms. MVT was in second position (21.1/100,000) and 30.2% of these victims were killed as pedestrians. For this age group, the risk of being a fatal victim of homicide was twice the risk of being a fatal MVT victim.

The fourth age group consisted of persons aged 45 to 59 years. The overall mortality rate was 79.5/100,000. The intent and mechanism analysis showed a pattern differing from what was found for the combined age group from 15 to 44 years. Among the deaths in this fourth group, the unintentional component accounted for a higher proportion than did the intentional component (49.6% and 34.9%, respectively). The leading cause was MVT, at a rate of 23.5/100,000. Deaths as pedestrians accounted for 31.8% of MVT deaths. Homicides were in second position (20.9/100,000) and firearms were responsible for 55.0% of these deaths. The suicide mortality rate was 6.8/100,000, which was the highest rate of all the age groups.

The final group consisted of older adults, i.e. people aged 60 years and over. Although the proportion of deaths for this age group was not high (4.9% of total), the age-specific rate was 105.2/100,000, which was very close to the rate for adolescents and young adults. Most of these deaths were classified as unintentional (56.6%). In comparison with all other age groups, they showed the lowest proportion of intentional deaths (12.5%) and the highest proportion with undetermined intention (30.1%). The leading cause of death was MVT (25.9/100,000); deaths as pedestrians accounted for a high proportion of these victims (52.2%). This age group had the highest mortality rate due to falls, in comparison with all other age groups (20.4/100,000); this figure was 5.5 times higher than the overall rate.

The ages of 535 fatal injury victims were unknown. To fill out death certificates, official identification documents are needed. This group represented 1.7% of all deaths due to injuries. Among them, most were homicides (48.2% overall), followed by undetermined intent (23.2% overall).

### Comparison

When analyses are broken down into states or cities, it is difficult to make international comparisons, because it is more common to find data on whole countries. The matrix used in this report allows international comparisons. Table 2 showed data from some selected states in the United States, for 2001;^10^ two of them were selected because their rates were very close to the injury mortality rates for the State of São Paulo (they were the top two injury mortality rates among all the states in the United States). The distributions by intent and mechanism were very different from the rates for the State of São Paulo. In the United States, the unintentional component was predominant. Among the intentional deaths, suicides were the leading cause, except in the District of Columbia, where Washington DC is located. This comparison is limited because unadjusted rates were used.

## DISCUSSION

These results highlight the problem of injury mortality among people living in the State of São Paulo. The greatest percentage of deaths were classified as intentional (49.7%), of which homicides were 44.6% and suicides were 5.0%. Usually, among deaths due to injuries, the unintentional component predominates in developed countries; data from the United States for 2001 showed that 65% of injury deaths were classified as unintentional, while suicides were 20% and homicides were 13%.^10^ Although the intentional component predominated for overall population of the State of São Paulo, the proportion varied between the different age groups: children, pre-adolescents and the elderly were more likely to be victims of unintentional death. However, adolescents and young adults were at the highest risk of being a victim of an intentional death. The leading mechanism for unintentional injury was MVT, for all ages, and among intentional injuries it was homicide.

According to the World Health Organization, road traffic injury is a major global public health and development problem that will worsen in the years ahead if no significant steps are taken to prevent it.^3^ The present report showed that pedestrians are a high-risk group for fatal MVT injuries. They are the most vulnerable players in the traffic system. Data for Brazil as a whole has shown similar proportions.^5^ Also, in low-income and middle-income countries, a large proportion of the road crash victims are pedestrians and cyclists.^3^ The highest proportions of pedestrians as victims of road crashes have been found in countries in South Asia like Bangladesh (62%), India (55%) and Pakistan (50%).^11^ On the other hand, the leading casualties in most high-income countries are among vehicle occupants, who are the main focus of prevention policies. It is crucial to provide equal protection to all road users. There are many well-tested and cost-effective solutions for addressing this problem.^3^

Violence has become a major public concern for all Brazilian society. Over the period from 1980 to 2002, homicide rates more than doubled in Brazil.^12^ The present results showed that homicide had a large impact on people aged 15 to 59 years. Because firearms played an important role among these deaths, it is very important for gun control policies to be implemented in Brazil. A law regulating firearms, called the Disarmament Statute, was approved by the Brazilian Congress in December 2003, and this makes it illegal to carry and own firearms in this country, for everyone except the police and armed forces, and it also deals with international firearms trafficking.^12,13^ It is probably necessary to wait longer in order to evaluate the results from this law on homicide numbers. Violence is certainly a very complex problem, but it is not a hopeless one and many experiences around the world have shown that it is possible to prevent violence.^2^ As an example, the homicide rates in the municipality of Diadema (in the Greater São Paulo metropolitan region) have been decreasing over recent years, and the authorities attribute this to a law that was implemented in July 2002,^14^ which prohibited bars from selling alcoholic beverages after 11:00 p.m.^15^ Also in the city of São Paulo, the rates have been reducing since 2000.^16^ There are some hypotheses for this decrease in São Paulo, but further studies are needed in order to establish the reasons for this.

It is important to draw attention to the victims whose age group was unknown, because this problem is especially related to injuries. These 535 fatal injury victims accounted for 64.3% of all deaths for all causes in 2003, for which the victims were classified as being of unknown age. These individuals probably did not have identification documents and there were no families to claim their bodies, or else these bodies were so decomposed that was impossible for medical examiners to estimate their age. In addition, for homicide victims, it is possible that relatives or friends were afraid of becoming involved in a crime. Another reason could be the funeral costs, which are considered to be high for families of lower income levels.

Although SIM is considered to be a very good quality information system, especially in the State of São Paulo, where the coverage is very high, this study presented some limitations. First, the proportion of unspecified injuries were very high (21.1% overall; 16.9/100,000). Thus, the analysis would have been more precise and would have brought more detailed information if this proportion had been lower. Among the elderly, 30.1% of the deaths due to injury were classified as undetermined intent. It may sometimes be difficult to determine whether an injury was accidental or intentional when it occurs to an older adult living alone.

Nonetheless, it is recommended that the quality of mortality information in the State of São Paulo should be improved. The Forensic Institute will play an important role in this. Some worthwhile activities would be to promote training on how to fill out death certificates better, or to provide medical examiners with more information from police records.

## CONCLUSIONS

These data indicate that there is a need to develop injury prevention strategies, in view of the magnitude of the problem. The intent and mechanism of these injuries vary according to age group. Homicides among young people have to be addressed. Motor vehicle traffic injuries play an important role for all ages.

Injury mortality rates reflect the most serious facet of the problem, but it is a small one. There is also a need to analyze the data from hospital admissions and emergency services in order to obtain an overall picture of the problem. If Brazilian society and public health professionals can envision living in a safer and more peaceful country, it is crucial to act together with other sectors from government and non-governmental organizations, to build appropriate and effective solutions that fit our needs.
